# Web-based interventions for fear of cancer recurrence: A scoping review with a focus on suggestions for the development and evaluation of future interventions

**DOI:** 10.1371/journal.pone.0312769

**Published:** 2024-11-08

**Authors:** Solveiga Zibaite, Sheela Tripathee, Helen Moffat, Beatrix Elsberger, Sara Maclennan

**Affiliations:** 1 Department of Psychological Sciences and Health, University of Strathclyde, Glasgow, United Kingdom; 2 Academic Urology Unit, School of Medicine, Medical Sciences and Nutrition, University of Aberdeen, Aberdeen, United Kingdom; 3 NHS Grampian, Aberdeen Royal Infirmary, Foresterhill Site, Aberdeen, United Kingdom; 4 NHS Grampian, Aberdeen Royal Infirmary, Breast Unit, Aberdeen, United Kingdom; University of Sharjah College of Health Sciences, UNITED ARAB EMIRATES

## Abstract

**Purpose:**

The objective of this scoping review is to provide an overview of the available evidence on the effectiveness of web-based interventions for fear of cancer recurrence (FCR) and a discussion of drawbacks and possible improvements for web-based interventions identified in the reviewed studies. These steps fulfil the aim of this review, which is to offer suggestions for developing future web-based interventions based on the reviewed studies.

**Methods:**

**Five databases (**PubMed, MEDLINE, EMBASE, SCOPUS and Web of Science**) were searched.** Original peer-reviewed articles, written in English, on web-based interventions for FCR were included for review. The data from the included studies was synthesised thematically.

**Results:**

We included 34 papers reporting on 28 interventions. Most of the studies in the papers were quantitative and mixed quantitative studies with a qualitative element, e.g. an interview post-intervention. Interventions were most commonly trialled with women breast cancer patients. Top three countries where studies were conducted were USA, Australia and the Netherlands. The most common theoretical framework for interventions is cognitive behavioural therapy (CBT), followed by mindfulness-based and mixed CBT, mindfulness, acceptance and commitment therapy (ACT), relaxation approaches. FCR was the primary focus/measure in 19 Studies, in 9 studies FCR was a secondary/related outcome/measure. Overall, the evidence of efficacy of web-based interventions on FCR is mixed.

**Conclusions:**

The existing research suggests several key points for producing more robust evidence about the effectiveness of web-based interventions for FCR. First, the studies suggest that it is a priority to better define eligibility criteria to proactively include people with higher levels of FCR. Second, there is a need for longer-term follow-up and outcome measuring period. Third, research examining the reasons for dropout from web-based interventions for FCR is critical to improve the effectiveness of web-based interventions. Fourth, while web-based interventions do not involve the costs of transportation, traveling time, space, equipment, cleaning, and other expenses, further cost utility analyses should be performed. Finally, future studies should assess how intervention accessibility, adherence, and effectiveness can be improved across different intervention designs, varying from intensive synchronous individual therapist-assisted web-based programme to blended designs combining the advantages of face-to-face and internet-based elements, to entirely self-managed programmes.

**Implications for cancer survivors:**

Developing and evaluating more accessible FCR treatments have been identified among top international FCR research priorities (Shaw et al. 2021). While there is some evidence that web-based interventions can be as effective as face-to-face interventions, currently there is a dearth of systematic data about the ways in which the web-based modality specifically can enhance supportive care for FCR. Developing knowledge about effective web-based interventions has implications for cancer survivors as they can be presented with more accessible, low-cost and low-burden options for managing fear of cancer recurrence.

## Introduction

Fear of cancer recurrence (FCR) is defined as ‘the fear, worry or concern relating to the possibility that cancer will come back or progress’ [1]. Contemporary debates on FCR centre around determining the threshold of clinically significant FCR [2], differences between fear of cancer recurrence and fear of cancer progression [3], development and refinement of new scales for measuring FCR [4], and calling for specific clinical guidelines to manage FCR [5]. The call for guidelines specifies that there is a need to clarify whether FCR care should be matched to the severity of FCR (i.e., nonelevated, elevated, clinically elevated) [5].

Supportive care for FCR is the most commonly reported unmet need of cancer survivors [6, 7]. Despite reported high FCR prevalence within various groups (ranging from 39% to 97% in all continents and at all time points since diagnosis [8]), not enough is known about what works best, who would benefit most from FCR interventions and at what points in the cancer pathway to introduce them. There are a number of existing systematic and metareviews on web-based interventions for cancer care/aftercare [9–17] and on fear of cancer recurrence interventions more generally [18–21]. Several psychological interventions have been developed to reduce FCR with varying levels of effectiveness [10, 19]. However, more easily accessible synthesised evidence is needed to understand the role of online interventions as tools for clinicians to address FCR at pertinent timepoints for patients.

This has been especially pertinent since the Covid-19 pandemic laid bare the devastating effects of limited access to usual face-to-face care and social isolation on health anxiety [22, 23] and FCR [24]. As such, it engendered a push to utilise the technological capabilities for web-based interactivity to improve health service delivery and in turn, patient wellbeing [25–27] Developing and evaluating more accessible FCR treatments has been identified as top international FCR research priorities [28] and there is some evidence that online delivery of interventions may be as effective as face to face interaction [29]. However, it has been noted that simply converting proven face-to-face interventions to eHealth delivery does not exploit the full potential of technological options [12] This means that online interventions should be considered to have unique functionality, accessibility and longevity aspects that require closer investigation in order to enhance their applicability and benefits to people experiencing FCR.

Our review addresses a gap in literature in four ways: 1) it focuses exclusively on web-delivered interventions with a focus on FCR regardless of their psychological framework, 2) presents the existing evidence on outcomes of studies on interventions for various levels of FCR severity, 3) addresses not only which psychological frameworks for FCR interventions are effective for reduction of FCR but also whether there is any variability in the suitability of these psychological frameworks (e.g. CBT, ACT, mindfulness, etc.) for web-delivery; and 4) highlights the unique aspects of online intervention design and delivery that might enhance their applicability in supportive cancer care.

There are various definitions of the modes of delivery of the interventions that, broadly speaking, are not face-to-face, such as web-based, remote, digital, mHealth, eHealth, etc. Consistent definitions and continuous developments of classification of these interventions is increasingly important as these various modalities of interventions proliferate. Notable attempts are made to do so by Matis et al. [12] and Skrabal-Ross et al. [13]. The focus of our review is web-based interventions for FCR. Our definition of web-based interventions includes both synchronous (interactive meetings with nurses, physicians and peers) and asynchronous (pre-recorded talks, self-guided information websites, downloadable digital material, and smartphone apps) modes of delivery of the intervention via a digitally enabled and interned aided platform. We will include blended interventions (face-to-face sessions in combination with online delivery of material and/or telehealth sessions), with a view of discussing the added benefits and arising challenges of the digitally enabled component of the intervention.

### Objectives

The objective of this scoping review is to provide an overview of the available evidence on the effectiveness of web-based interventions for FCR and a discussion of drawbacks and possible improvements for web-based interventions identified in the reviewed studies. This helps achieve the overall aim of the review which is to offer suggestions for developing future web-based interventions based on the reviewed studies

## Methods

This is a scoping review conducted building on Arksey and O’Mally’s framework [30] to map the existing literature in a field of interest in terms of the volume, nature, and characteristics of the primary research. The main reviewer (SZ) performed an initial literature search on Pubmed, EMBASE, Web of Science, SCOPUS and MEDLINE databases in June 2024. Search terms associated with (1) FCR, combined with search terms associated with (2) cancer and (3) web-based intervention were used. The full search query can be found in the S1 Appendix. The reference lists of identified papers were also reviewed to identify further relevant sources that might have been omitted in the initial search. We report this review based on the Preferred Reporting Items for Systematic reviews and Meta-Analyses extension for Scoping Reviews (PRISMA-ScR) Checklist, which can be found in S1 Checklist.

### Inclusion and exclusion criteria

**Fig 1:**
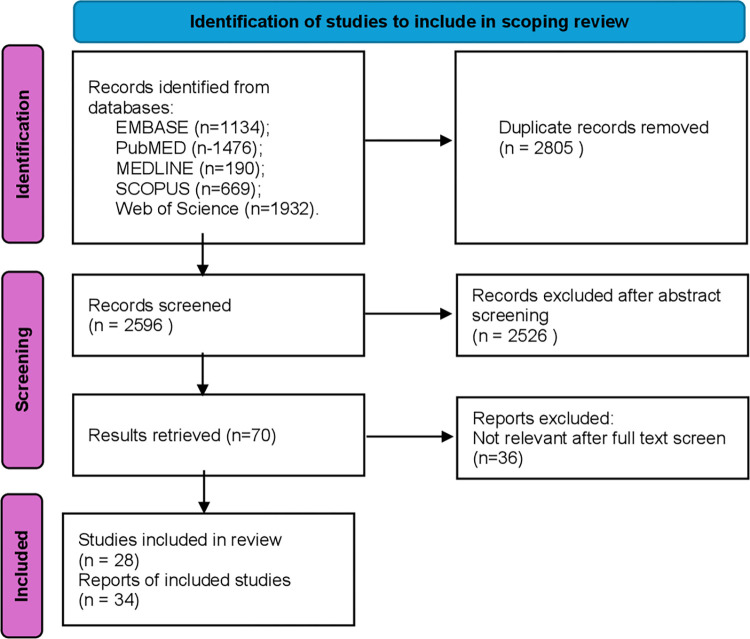
Literature search flowchart.

The search aimed to identify English language (1) peer-reviewed (2) literature reporting data from qualitative, quantitative and mixed-methods studies concerning the creation, delivery, or evaluation of a web-based intervention where reduction of FCR is a primary or secondary outcome measure (3) in people (both children and adults) who have been diagnosed with cancer. There was no restriction on cancer type, cancer stage, time since diagnosis, treatment type, or country/region of residence. We included papers based on primary and secondary analysis of research data. The literature search included studies published until June 2024, without a date cut-off for the earliest publication. This was suitable because of the relative recency of the emergence of interest in FCR as an onco-social phenomenon and the proliferation of digitally enabled interventions. All eligible articles were included in the review regardless of quality assessment rating as assessment of the quality of studies does not form part of the scoping review remit [30]. Excluded studies: not-peer reviewed, commentary, editorials and opinion paper, conference abstracts, study protocols, studies not in English language. The literature search flowchart can be found in Fig 1.

### Data extraction and synthesis

Data was extracted using the SPIDER framework (sample, phenomenon of interest, design, evaluation and research type) [31]. For each study, the following information were extracted: author, year of publication, country, study design, sample size, cancer type, sex of participants, FCR measure, intervention features. A full text reading of each paper also allowed to pull out the key findings and messages regarding the value and pitfalls of using online mode of delivery for the interventions. The extracted data and overall text were analysed using a thematic analysis framework [32]. The final set of themes was reviewed and determined by all members of the research team.

## Results

A total of 34 papers reporting on 28 different interventions are included in this review. All of them were identified first in the database searches and no new studies were identified in the manual reference list search.

### Study characteristics

Of the included interventions, 15 were tested using a randomized controlled trial (RCT) design [19, 33–46] and 10 [47–56] were variations of development, pilot feasibility, usability and satisfaction studies. We also included one qualitative study about cancer survivors’ experience of participating in a virtual cognitive behavioural therapy-based telephone coaching program for fear of recurrence, a Canadian government funded BounceBack program [57]. One was a prospective observational study [58] based on a secondary analysis of trial data, and one was a quasi-experimental mixed-methods study [59]

Out of the 15 RCTs, twelve [33, 34, 37, 39–42, 44–46, 60, 61] had a no-intervention control (treatment as usual (TAU)). One out of these twelve studies, one [34] had an additional active intervention arm–face-to-face mindfulness-based cognitive therapy (MBCT) program. Two studies [35, 36] had a control condition of a slightly altered tested intervention. In FoRtitude trial [43],the control group received health management content.

Out of the 10 feasibility studies, [47, 49, 50, 52–56, 62] nine were a single-group, pretest-post-test pilot and one was a quasi-experimental effectiveness, acceptance and satisfaction study [59]. The aforementioned quasi-experimental study had a treatment as usual control arm but had to omit randomisation due to a small sample of participants.

Most of these studies were mixed quantitative studies with a qualitative element, e.g. an interview post-intervention. Most common countries for the studies were Netherlands (n = 6), Australia (n = 6) and USA (n = 5). The rest of the studies were based in Germany (n = 3), Taiwan (n = 1) and Japan (n = 2), UK (n = 1), Canada (n = 1), Switzerland (n = 1), China (n = 1), Denmark (n = 1). The characteristics of the included studies are presented in Table 1.

**Table 1:** Included study characteristics.

	Study or intervention name; Articles: authors, year of publication, country	Study type	Sample size, gender, mean age	Cancer type/stage	Theoretical framework/ base for content	Intervention delivery	Duration, frequency
**1**	**BREATH** van den Berg et al. (2015), Netherlands [33]	RCT	150 females mean age 50.18)	Breast cancer, completed curative intent treatment	CBT and psychoeducation	Self-managed website on early survivorship (information, homework, assessment with automated feedback)	16 weeks, weekly modules.
**2**	**BeMind**	RCT	245: 210 females, 35 males. Mean age 51.7.	Various cancers; stages 1–4; receiving/after primary treatment	Mindfulness-based CBT	Website, emails with therapist, audio recording, daily meditation exercises.	Eight weeks, weekly modules, weekly emails with therapist.
	Compen et al. (2018); [29]						
	Bisselling et al. (2018); [63]						
	Cillessen et al. (2020), Netherlands [64]						
**3**	**Gratitude-eliciting writing intervention** Otto et al. (2016), USA [35]	RCT	67 females, mean age 56.89	Early-stage breast cancer (defined as Stage 0 [ductal/lobular carcinoma in situ], Stage I, Stage II, or Stage IIIA)	Positive psychology, gratitude	Self-managed, web-based delivered prompts to write a letter of gratitude to a person of the participants’ choice, weekly questionnaires	Six weeks, 10 minutes per week + time for questionnaires.
**4**	**AIM-FBCR** Lichtenthal et al. (2017), USA [36]	RCT	110 females, mean age 55.8	stage 0 to stage III breast cancer with no history of disease recurrence or metastases and who completed active treatment 3 months prior	Cognitive bias modification	Individually tailored computer delivered cognitive bias modification exercises	Four weeks, eight sessions (twice a week), 30 minutes each.
**5**	**SWORD** van de Wal et al. (2017); [37]	RCT	88. 41 males, 47 females. mean age for 58	Various cancer survivors from 6 months to 5 years after cancer treatment	Blended CBT	Blended intervention: face-to-face sessions, combined with three 15-minute e-consultations	12 weeks
	Burm et al. (2019), Netherlands [38]						
**6**	**MindOnline** Russell et al. (2019), Australia [39]	RCT	69 (mean age 53.4, 54% female)	a melanoma diagnosis of stage 2c, 3a, 3b or 3c, completed their last treatment within the past 5 years	Mindfulness	Self-guided website (short videos, guided mediations)	Six weeks, weekly modules
**7**	**CAREST (Less fear After Cancer)** van Helmondt et al. (2020), Netherlands [40]	RCT	262 females, mean age 55.8	diagnosis of breast cancer 1 to 5 years ago, no signs of local or regional recurrence or metastatic disease	CBT	Self-guided website. Email coach available for people who exhibited a need for it.	4–6 weeks course
**8**	**Healthy living with breast cancer (SCP-A)** Fang et al. (12020), [41] Taiwan	RCT	165 females, mean age 51.55	Diagnosis of breast cancer, having completed their primary treatment but less than 5 years without a sign of recurrence	Survivorship education	Survivorship care plan computerized app (text and videos)	Five weeks, seven modules
**9**	**iCanADAPT-Early** Murphy et al. (2020), [42]	RCT	114 (101 females, 13 males), mean age 53.29	Mixed various stage cancers	CBT	Self-managed, clinician supervised website (text, video, audio, homework)	16 weeks, eight lessons
	Davies et al. (2022), Australia [65]						
**10**	**FoRtitude** Wagner et al. (2021), USA [43]	RCT	196 females, mean age 54.7	Stage 0-III breast cancer at diagnosis, completion of primary breast cancer treatment 1–10 years before consent (current hormonal treatment allowed), disease free	CBT	eHealth website with didactic content, interactive tools and interactive messaging feature, also tele coaching.	Four weeks, website updated three times a week, four weekly telephone calls,
**11**	**My-GMC** Visser et al. (2018), Netherlands [44]	RCT	109 females, mean age 55.8.	Breast cancer diagnosis, primary treatment completed at most 5 years ago	Group medical consultations and tablet-based web-based support group sessions	Video conferencing and tablet-based web-based app (videos, survivorship information) and three web-based support sessions.	Three group meetings over three months, 60 minutes each.
**12**	**Online mindfulness based intervention.** Peng et al. (2022), China [45]	RCT	60 females, aged 18–65 years	Stage I-IV breast cancer diagnosis, completion of all treatments with the exception of hormonal or Herceptin therapy and no cancer recurrence or metastasis	Mindfulness-based course	Online course delivered in group sessions; assignments for home practice (30 mins per day) accompanied by the recordings of 5P medical app (an app to promote mind and brain health and cultivate happiness)	Six weeks, once weekly group sessions, 1,5 h each.
**12**	**ConquerFear‐Group.** Tauber et al. (2023), Denmark [60]	RCT	85 females, mean age 54.5.	Breast cancer survivors who had completed primary treatment 3 months–5 years previously, were ≥18 years, and scored ≥22 on the Fear of Cancer Recurrence Inventory–Short Form (FCRI‐SF).	Sessions focusing on metacognitive strategies, values‐ clarification, and education about follow‐up behaviour).	Online delivered group sessions	Six weeks total—one 1½ hour individual session followed by five weekly 2‐h group sessions. Home exercises and reading materials.
**14**	**Kaiketsu app and Genki app (Genki means energy or vitality in Japanese,** (Akechi et al. 2023), Japan [46]	RCT	447 females, median age 45.	age 20–49 years, 1 year after breast surgery, currently disease-free.	Kaiketsu app problem solving therapy. Genki app–behavioural activation	The Kaiketsu-App comprised nine sessions. Each session took approximately 10 minutes to complete. The Genki-App comprised six sessions, with approximately 10 minutes needed to complete each session.	8 weeks
15	**“Less fear after cancer”—Guided online primary care intervention** (Luigjes-Huizer et al 2023), Netherlands [61]	RCT	167 participants (131 women and 36 men) mean age 53.3.	adult (≥18 years) cancer survivors who had (a)finished successful curative cancer treatment between 3 months and10 years ago, (b) wanted support for FCR, and (c) had sufficient Dutch reading and writing skills.	cognitive behavioural therapy and the model on FCR by Lee-Jones(1997)	Information videos, exercises, three to five 30-min video calling sessions with a mental health worker	10 weeks
**16**	**Onco-STEP** Seitz et al. (2014), Germany [56]	Feasibility and satisfaction study	20 (14 female, 7 male), mean age 27.25.	Former patients of pediatric cancer, older than 15 years manifesting clinically relevant PTSS or anxiety were eligible	CBT	Website with two modules and ten writing assignments, ability to communicate with participants and therapist via asynchronous chat.	Five-six weeks
**17**	**e-TC** Heiniger et al. (2017) [47], Australia	Development and pilot testing study	25 males, mean age 37.6.	Testicular cancer, treatment completed between six months and five years previously.	CBT, ACT, metacognitive therapy, mindfulness and relaxation	Self-guided website (psychoeducational material, videos, offline exercises)	Six weeks, six modules, one hour each.
**18**	**e-TC 2.0** Smith et al. (2020), Australia [48]	feasibility, acceptability and preliminary efficacy study	39 males, mean age 37.1 years.	Finished active treatment for testicular cancer, currently disease-free, above clinical cut-offs on the HADS (≥8) and/or the FCRI-SF (≥13).	Same as e-TC	Same as e-TC but with improvements identified in the pilot study: 1. Quick access to relevant content via bookmarking; 2. More interactivity; 3. Increased video content.	Six weeks, six modules, one hour each.
**19**	**mBBSR(BC)** Lengacher et al. (2018), USA [49]	Single‐group, pretest‐posttest pilot study	15 females, mean age 57.	breast cancer; stages 0–3; primary treatment completed	Mindfulness-based stress reduction	Tablet-based mobile app (video, audio, booklet, meditation), some contact with clinical psychologist.	Six weeks, six sessions (120 mins each), once weekly telephone calls.
**20**	**Kaiketsu-app** Imai et al. (2019), Japan [50]	Feasibility and preliminary effectiveness study	38 females, mean age 44	Diagnosis of invasive breast cancer, currently disease-free; had breast surgery over 6 months ago.	Problem- solving therapy	Smartphone app, phone and email contact from research team.	Eight weeks.
**21**	**iConquearFear** Smith et al. (2020) [62]	Development and usability study; feasibility and preliminary efficacy study	23 (19 female, 4 male), average age 53 years.	Treatment for melanoma, breast cancer, colorectal cancer, ovarian cancer, or prostate cancer completed with no evidence of recurrence	Metacognitive therapy and ACT	Self-guided website (audio, video, text)	Two weeks.
	Smith et al. (2022), [51] Australia						
**22**	**iHOPE** Martin et al. (2020) [52], UK	Pre, post acceptability and feasibility study	114 (102 females, 12 males), mean age 51.3	Various cancers, patients coming to the end of cancer treatment or surgery, or having recently completed treatment.	Hope therapy, positive psychology and CBT	Peer delivered, largely self-managed website (text, interactive activities, downloadable material)	Six weeks
**23**	**Virtual resiliency program for lymphoma survivors** Perez et al. (2021) [53], USA	Feasibility, acceptability, and preliminary efficacy study	26, 50%male, 50% female, mean age 52.4	Lymphoma patients who were within two years of completing treatment for lymph	Stress-coping skills grounded in mind-body, CBT and positive psychology.	Group videoconferencing	Eight weeks, weekly sessions
**24**	**Online booklet for ovarian cancer survivors** Pradhan et al. (2021) [54], Australia	Acceptability and satisfaction study	62 females, mean age 56.9	Ovarian cancer diagnosis	FCR management information, self-help	An online booklet	-
**25**	**Mindfulness and relaxation app** Mikolasek et al. (2022) [55], Switzerland	Mixed-methods feasibility study	100 patients (74 female) mean age 53.2	Breast, colorectal, prostate cancer patients; stages 0–4; receiving/after primary treatment;	Mindfulness-based eCBT	Self-guided mobile app with audio recordings	Ten weeks
**26**	**Web based chat group for prostate cancer patients** Lange et al. (2017) [59], Germany	Quasi-experimental mixed methods study	44 males, mean age 60.5	Diagnosis of prostate cancer	Peer support	Online chat program, guided by psychotherapists.	Five weeks, weekly sessions.
**27**	**BounceBack** Nguyen et al. (2022) [57], Canada	Mixed-methods study exploring the impact of virtual CBT-based support on psychosocial symptoms	44 females, mean age 57.	Various cancers, after finishing treatment	CBT	Online workbooks and telecoaching	Maximum of six months
28	**Video sequence based intervention** (Schlect et al. 2023) Germany [58]	Prospective observational study	155 patients, (56.1 years)	malignant tumor disease, 18-years or olde/	PST and BA (behavioural activation)	Website where two 12–30 minute long videos prepared by an psycho-oncologist provided weekly.	4 weeks

From the extracted data we identified six key themes that will be presented in this review: FCR measures used, considerations about target populations for web-based intervention; technological challenges; improving adherence; implementing interventions in existing pathways and choosing a theoretical framework for interventions. The following discussion is structured thematically.

### Population characteristics

The population characteristics of the included studies are reported in Table 1. Most of the studies focused on people that had had cancer and were currently free of disease (n = 21). Seven [34, 35, 54, 55, 57–59] studies involve participants who are currently living with cancer. Most studies had female only participants (n = 15) followed by both male and female (n = 11) and two studies had only male participants [47, 48]. Most studies focused on patients with breast cancer (n = 12), followed by patients affected by various cancers (n = 10), testicular cancer (n = 2) and pediatric cancers (n = 1), lymphoma (n = 1), melanoma (n = 1), ovarian cancer (n = 1). All studies with breast cancer as their focus had only female participants. In studies where patients affected by various cancers were included, females were significantly overrepresented (76% of participants). Only one study [53] had equal numbers of male and female participants.

The language of the interventions depended on the patient population geographical location general spoken language. 13 interventions were in English [35, 36, 39, 42, 43, 47–49, 52–54, 57], Two were in Japanese [46, 50], 6 were in Dutch [33, 34, 37, 40, 44, 61]. Two were in Chinese [41, 45], four were in German [55, 56, 58, 59], one was in Danish [60].

### FCR outcome

FCR was the primary focus/measure in 19 studies [35–40, 43, 45, 46, 50, 52, 54–57, 60–62], in 9 studies FCR was a secondary/related outcome/measure [33, 34, 41, 42, 44, 47, 49, 58, 59].

### FCR measure

In terms of measuring fear of cancer recurrence, in our reviewed studies the FCRI scale was the most widely used (n = 8) [34, 37, 39, 42, 43, 57, 60, 61]. FCRI is a popular 42-item multi-dimensional FCR measure with strong psychometric properties. Also used were the brief FCR measures FCRI-SF (n = 3) [45, 51, 60]; FCRI—SF- NL (n = 1) [40]; Four item FCR scale (n = 1) [47]. FCRI and its short form were popular due to being measures created in USA and also prevalent in Australia, where most of the studies originated from. Other measures used were Concerns About Recurrence Scale (CARS) (n = 4) [35, 36, 49, 50]; Cancer Worry Scale (CWS) (n = 3) [33, 37, 44]; Japanese version of the Concern About Recurrence Scale (CARS-J) (n = 2) [46, 50]; Short form of the Fear of Progression and Relapse Questionnaire (FOP-SF) (n = 4) [54–56, 58], Two subscales of the Quality of Life of Adult Cancer Survivor Scale (QLACS) that specifically address frequently cited unmet needs for survivors: cancer-related fatigue and cancer-related concern or fear of recurrence were used in one study [52]. One study used the German version of the MAX-PC [59].

Perez et al. [53] paper on survivorship education plan for lymphoma survivors did not have a FCR specific measure assessing more general cancer-related uncertainty. Perez’s study was included in this review because the participants in Perez’s study raised the importance of fear of cancer recurrence being addressed and the interviews revealed how the remote delivery of a program may aid that.

### Screening for eligibility

Only 7 reviewed studies used FCR measures for eligibility to participate in the studies. FoRtitude trial [43] included those with score 13 and over on the FCRI severity subscale. SWORD [37] included people scoring >14 on CWS, 6 month to 5 years after cancer treatment. BeMind [34] recruited people with a score of >11 on the Hospital Anxiety and Depression Scale (HADS). iCanADAPTEarly study [42] used HADS Total score of ≥6. Lichtenthal et al. [36] included breast cancer survivors who scored >3 on the CARS Overall Fear Index. ConquerFear-Group included those who scored ≥22 on the FCRI-SF [60].

Out of the studies that did not screen for FCR as part of eligibility criteria, only the following contained some note of justification about doing so. Heiniger et al. [47] piloted the e-TC intervention with testicular cancer survivors without significant psychological morbidity and later piloted an updated intervention (e-TC 2.0) [48] with participants with borderline/clinical levels of anxiety, depression, and/or FCR based on their pilot findings that the intervention is more beneficial for individuals who exhibit heightened FCR. In a study that has not screened for FCR [47], authors speculated that participants, exhibiting higher the clinical need for support were more likely to adhere to and complete the programme as well as report greater effects. The authors of the iConquerFear study [62] justified their lack of FCR screening because the FCR prevalence in their target populations (breast, colorectal, melanoma, prostate and ovarian cancers) was high. Otto et al. [35] noted that the level of wellbeing participants in their trial exhibited at the pre-assessment—low levels of anxiety, depression, and physical symptoms–was not necessarily representative of all early-stage breast cancer survivors. Related to this, Van Helmondt et al. [40] made a note that their CAREST trial intervention had ‘high ecological validity because not screening on level of FCR, the use of CAU (care as usual) and self‐help without extra help or emails reflected a realistic picture of web-based self‐help interventions’. This is a valid point for evaluating the status quo of the web-based self-help intervention landscape, but we argue it does not hold up against the aim of developing, trialling and evaluating future interventions to address unmet FCR needs. All in all, these results show that the included interventions have been tested with participants exhibiting a wide range of FCR severity.

### Theoretical framework

Various theoretical frameworks were used for building the interventions. The most common theoretical framework for interventions was cognitive behavioural therapy (CBT) (n = 12) [33, 36, 37, 40, 42, 43, 50, 56, 57, 60, 62]. Second most common framework as mindfulness-based approaches (n = 4) [39, 45, 49, 55], such as mindfulness- based cognitive therapy [34]. Two studies were based on problem-solving therapy [46, 50] and behavioural activation [46], one study was a gratitude-eliciting intervention [35]. Four studies used mixed approaches: a combination of CBT, ACT, metacognitive therapy, mindfulness and relaxation [47, 48]; positive psychology, CBT and hope therapy [52]; mind and body, cognitive behavioural and positive psychology principles [53] The rest of the interventions reviewed built on more information-based approaches: FCR management information (n = 1) [54], a survivorship care plan (n = 1) [41]; group medical consultations in combination with a peer support app (n = 2) [44, 53].

### Fear of recurrence intervention feasibility findings

All the interventions in this review evaluated in feasibility and usability studies have been deemed feasible and acceptable (n = 10).

### Fear of cancer recurrence intervention effectiveness findings

Out of 15 included randomised trials, eleven showed effectiveness [33–37, 39, 41, 42, 45, 46, 60]. We cannot compere these as the studies used different measures. The sustainability of these effects was variable. Fang et al.’s online survivorship care plan [41] computerized application (SCP-A) was effective in decreasing the participants’ fear of recurrence after 12 months, and the effects of Luigjes-Huizer et. al’s primary care setting FCR intervention were visible after 10 months [61]. While Lichtenthal et al.’s cognitive bias modification intervention Attention and Interpretation Modification for Fear of Breast Cancer Recurrence (AIM-FBCR) [36] showed preliminary effectiveness in reducing FCR in survivors of breast cancer and levels of worry in women who received the intervention decreased from high-moderate to less than moderate, the CARS Health Worries subscale was the only one that remained clinically significantly changed after three months post-intervention [37]. Van den Berg et al. [33] reported that participants in BREATH programme together with care as usual showed a greater decrease in fear of cancer recurrence, fatigue, and general and cancer-related distress. SWORD study found to significantly reduce long-term (15 weeks) FCR [37, 38]. Compen et al. [34] found that compared with TAU, both MBCT and eMBCT significantly reduced fear of cancer recurrence. In the long term, the reduction of psychological distress was significantly higher in eMBCT than in MBCT [64]. Interestingly, Luigjes-Huizer et al. [61] found that while FCR severity decreased and participants were generally satisfied with the outcome, many participants still scored above the FCR cutoffs. The authors 13 (87%) and 16 cut‐offs (74%).

CAREST trial demonstrated no effect of CBT‐based online self‐help training to reduce FCR in breast cancer survivors compared with CAU (after 3 and 9 months) [40] and two years on [66]. The efficacy of FoRtitude, a CBT-based eHealth intervention specifically targeting FCR, could also not be proven effective by the authors because FCR decreased significantly across the attention control (health management content) as well [43]. Wagner et al. (2021) speculated that in this trial, eHealth delivery may be responsible for the lack of main effects between CBT and HMC (health management content) They believed that their eHealth platform might have diluted intervention effects. However, the authors do not provide the level of detail needed to justify this conclusion.

The feasibility and satisfaction studies in our review that showed the highest preliminary effectiveness were Onco-step [56], Kaiketsu-app [50, 58] and BounceBack [57]. The authors of these studies provided different accounts of their success. Imai et al. [50] developed their intervention specifically for smartphone use with a view of improving feasibility and portability, and to in part avoid the challenges associated with other remote web-delivered interventions–being time consuming and restrictive in terms of location (in the web-delivered case–desktop or laptop as not everyone carries them around). Seitz et al. [56] at the time had developed a broad scope therapist-guided intervention that would address post-traumatic stress symptoms, general anxiety, fear of recurrence, and promote the AYA survivors’ self-efficacy and independence, where the internet aspect also comes into play as it is a familiar mode of communication for young adults. Nguyen et al.’s [57] virtual CBT-based intervention responded to their identified need for an accessible, less-labour intensive for patients and less of a burden on the healthcare system mental health service and resource for post-treatment and survivorship cancer programs and clinics. This study also targeted and measured three psychosocial symptoms—depression, anxiety, and fear of recurrence.

On the other side of the spectrum of effectiveness, Pradhan et al.’s e-booklet [54], based on the Conquerfear program [67], did not significantly improve levels of FCR in women with ovarian cancer. No significant changes in FCR were reported in Mikolasek et al. study evaluating the feasibility and satisfaction of a mindfulness app [55]. Lange et al. [59] found that intervention participants reported poorer results for the primary and secondary outcomes in comparison to the control group patients at follow up. A secondary analysis of an RCT that showed no significant effects on anxiety, depression and fatigue [58], showed that there were small changes in the severity of FCR but because of the observational design feature of the study it was not possible to attribute these effects to the intervention.

Overall, while all the included interventions had been deemed feasible and acceptable, the evidence on preliminary effectiveness were mixed and requires further investigations in larger trials. The evidence from randomised control trials included in this review were more uniform but requires longer follow ups to examine the sustainability of the effects and further investigation into how the online modality specifically affects the intervention effectiveness outcomes.

### Mode of delivery

The most popular mode of delivery across our reviewed studies was creating a dedicated website that hosted intervention material (n = 13) [33, 34, 39, 40, 42, 43, 47, 52, 56–58, 61, 62]. Two studies were tablet-based [44, 49]; two interventions were based on writing tasks [35, 56]; four studies reported development and evaluation of smartphone app-based interventions [41, 46, 50, 55]; four studies made use of telehealth group sessions [44, 45, 53, 60]; one study tested an online evidence-based psychoeducational booklet [54]; one study used individually tailored cognitive bias modification exercises [36]; one study utilised an online chat program [59]. The only intervention included in this review that still retained a significant face to face element was SWORD [37]. Three studies had phone communication as an additional element [43, 50, 57].

### Intervention frequency and duration

The duration of the interventions reviewed ranged from a one-ff reading an online psychoeducational booklet with a follow-up one week later [54] to a 16-week program [42, 33]. The most common duration of the intervention was 6 weeks (n = 9) [35, 39, 45, 47–49, 52, 60] followed by 8 weeks (n = 4) [34, 46, 50, 53], 4 weeks (n = 3) [36, 43, 58], 10 weeks (n = 2) [55, 61], 12 weeks (n = 1) [37], 5 weeks (n = 2) [41, 59]; 16 weeks (n = 2) [33, 42]; 2 weeks (n = 1) [62]. Two studies provided a range instead of precise duration: 4–6 weeks (n = 1) [40] and 5–6 weeks (n = 1) [56]. 6 months was the expected time for BounceBack program completion [57]. My-GMC consisted of three group meetings over three months [44]. Wagner et al. [43] speculated that the FoRtitude trial did not yield evidence of efficacy of any of the FCR intervention components because of the low intensity and duration of the intervention (2 didactic lessons and a tool to use over 4 weeks). The CAREST [40] trial with 4–6 weeks required to complete all modules also did not report positive results. It had been suggested that longer duration of intervention would be more beneficial to patients with higher levels of FCR [10] however there was no consensus on the optimal duration and frequency of web-based interventions.

### Target groups for FCR interventions

In those interventions included in our review where participants were affected by various cancers, it was often claimed that the intervention content was relevant to all cancer survivors regardless of the type of cancer [34, 52, 62]. However, the burden of FCR might be higher in some survivor cohorts, e.g. those dealing with more aggressive, more commonly recurring cancers, such as lymphoma [53], or among younger people [56]. ‘Those trials that involved specific age populations (i.e. pediatric cancers), or specific cancer types (i.e. breast cancer, testicular cancer) had developed these interventions to address specific pertinent concerns for these populations, such as intimacy, aesthetics. However, across these studies it was acknowledged that the content can be easily tailored for other cancer populations, or adapted to be used across all cancers.

Nevertheless, sharing the same lived experience also helps build rapport and a sense of group connection. Thus, it is worth considering how can the interventions be tailored to respond to these specific needs as accurately as possible. Seitz et al. [56] speculated that the treatment protocol was too structured for the youngest pediatric cancer survivors participating as all of the non-completers were significantly younger than participants fully completing the intervention. The authors also considered that the young adults might have missed the more lay and open communication or the informal interaction with peers. Smith et al. [62] argued that internet-based interventions may be particularly applicable to FCR because younger age is associated with higher FCR and web-based self-management acceptability. However, older age was associated with greater iConquerFear engagement [51], which was consistent with Cillessen et al.’s [64] findings that old age was not a predictor of engagement, but a predictor of uptake. Increased digital literacy of older adults supports the relevance of web-based interventions for all age groups.

### Recruitment strategies

Only a minority of studies used one avenue of recruiting [33, 58, 60] and these were going through medical records [58, 60] and being referred onto trial by treatment team [33]. The other studies have used various combinations of recruitment strategies. Being referred onto trial by treatment team [33, 45, 55, 59, 67] was the most frequent employed strategy, followed by recruiting participants via social media [46, 55, 56, 61], cancer patient organisations [46, 54, 55, 61], reviewing medical records [57, 58, 60], exhibiting posters [46, 67], disseminating a leaflets [55]. Interventions where direct care team was involved in recruitment had better rates of adherence and completion, however that was also variable depending on the type of intervention (clinician managed or self-guided) so with this available evidence we are not able to currently establish a particular strong link between recruitment strategies and participant retention and completion.

We were able to observe that those interventions with less common cancer populations or more vulnerable participants employed more diverse recruitment strategies. For example, the former pediatric cancer patients for Onco-STEP [56] were recruited among participants in a former study on late psychosocial effects of adolescent cancer and by distributing information leaflets in hospitals, it was also advertised in newspapers and magazines, on Internet platforms, in broadcasts, and oral presentations at meetings of young adult cancer survivors. Parental consent was obligatory for applicants under 18 years of age, but there was otherwise no involvement from them.

### Benefits and drawbacks of web-based interventions: self-guided or clinician-led?

The general benefits of the web-based delivery method reported in the reviewed studies were: own time management regarding access to self-guided materials; the ability to access the intervention (synchronous or asynchronous) from a familiar home setting; providing social validation and access to psychosocial support without the burden of travel, missing work and arranging and paying for childcare. Participant reported drawbacks varied, but most prevalent complaint was lack of contact and interaction with clinicians and for some, peers. Other drawbacks reported were: intervention perceived as impersonal [57], preference for printed workbooks over the website [37], higher dropout rates [34], technical improvements like keeping on top of the browser updates [47], scheduling challenges [53]

Contact with those who deliver and facilitate the intervention was an important aspect in intervention design in our reviewed studies. Qualified therapists delivered the intervention in ten reviewed studies, most often alongside the website self-guided materials [37, 43–45, 53, 56, 57, 59, 61]. Clinician supervision to a lesser extent (e.g. asynchronous communication, providing feedback, monitoring, tele coaching if required [34, 40, 42, 49, 56] was present in four studies [34, 40, 42, 49, 56]. Research teams were involved to provide prompts to participate in the intervention in two studies [35, 50]. One intervention was facilitated by trained peer-facilitators who were affected by cancer in some way [52]. 10 interventions were entirely self-guided. [33, 35, 36, 39–41, 47, 51, 54, 55]. The vast majority of the studies took place in a mental health setting and Luighes-Huizer et al. (2023) were the first to evaluate the impact of a psychological FCR intervention in primary care [61].

Reviewed articles often discussed the benefits and drawbacks of providing entirely self-managed interventions and self-guided resources. Self-guided/self-managed interventions were lauded for maximising scalability and reducing the time burden on both patients and medical staff members [50], but on the other hand, the amount of time required to complete self-managed interventions was lamented by participants as one of the drawbacks [47] especially in those that require daily commitment [34, 35, 43, 49, 50, 56, 57]. s It is important to note that in some interventions that were as a whole self-managed, some clinician supervision and contact has been retained [42, 49, 50, 66]. This is summarised in Table 2. Table 3 details the nature and extent of contact with clinicians in clinician delivered interventions for comparison.

**Table 2:** Self-managed interventions—nature and extent of contact with clinicians.

**BREATH** van den Berg et al. (2015), Netherlands [33]	Self-managed website on early survivorship (information, homework, assessment with automated feedback)
**Gratitude-eliciting writing intervention** Otto et al. (2016), USA [35]	Self-managed, web-based delivered prompts to write a letter of gratitude to a person of the participants’ choice, weekly questionnaires
**MindOnline** Russell et al. (2019), Australia [39]	Self-guided website (short videos, guided mediations)
**CAREST (Less fear After Cancer)** van Helmondt et al. (2020), Netherlands [40]	Self-guided website. Email coach available for people who exhibited a need for it.
**Healthy living with breast cancer (SCP-A)** Fang et al. (12020), [41] Taiwan	Survivorship care plan computerized app (text and videos)
**iCanADAPT-Early** Murphy et al. (2020), [42]	Self-managed, clinician supervised website (text, video, audio, homework)
Davies et al. (2022), Australia [65]	
**Onco-STEP** Seitz et al. (2014), Germany [56]	Website with two modules and ten writing assignments, ability to communicate with participants and therapist via asynchronous chat.
**e-TC** Heiniger et al. (2017) [47], Australia	Self-guided website (psychoeducational material, videos, offline exercises)
**e-TC 2.0** Smith et al. (2020), Australia [48]	Same as e-TC but with improvements identified in the pilot study: 1. Quick access to relevant content via bookmarking; 2. More interactivity; 3. Increased video content.
**mBBSR(BC)** Lengacher et al. (2018), USA [49]	Tablet-based mobile app (video, audio, booklet, meditation), some contact with clinical psychologist (once weekly phone calls)
**Kaiketsu-app** Imai et al. (2019), Japan [50]	Smartphone app, phone and email contact from research team.
**iConquearFear** Smith et al. (2020) [67]	Self-guided website (audio, video, text)
Smith et al. (2022), [51] Australia	
**iHOPE** Martin et al. (2020) [52], UK	Peer delivered, largely self-managed website (text, interactive activities, downloadable material)
**Online booklet for ovarian cancer survivors** Pradhan et al. (2021) [54], Australia	An online booklet
**Mindfulness and relaxation app** Mikolasek et al. (2022) [55], Switzerland	Self-guided mobile app with audio recordings
**Kaiketsu app and Genki app (Genki means energy or vitality in Japanese,** (Akechi et al. 2023), Japan [46]	The Kaiketsu-App comprised nine sessions. Each session took approximately 10 minutes to complete.
	The Genki-App comprised six sessions, with approximately 10 minutes needed to complete each session.
**Video sequence based intervention** (Schlect et al. 2023) Germany [58]	Website where two 12–30 minute long videos prepared by an psycho-oncologist provided weekly.

**Table 3:** Clinician delivered interventions–nature and extent of contact with clinicians.

Study or intervention name; Articles: authors, year of publication, country	Intervention delivery
**BeMind** Compen et al. (2018); [29]	Website, emails with therapist, audio recording, daily meditation exercises.
Bisselling et al. (2018); [63]	
Cillessen et al. (2020), Netherlands [64]	
**AIM-FBCR** Lichtenthal et al. (2017), USA [36]	Individually tailored computer delivered cognitive bias modification exercises
**SWORD** van de Wal et al. (2017); [37]	Blended intervention: face-to-face sessions, combined with three 15-minute e-consultations
Burm et al. (2019), Netherlands [38]	
**FoRtitude** Wagner et al. (2021), USA [43]	eHealth website with didactic content, interactive tools and interactive messaging feature, also tele coaching, four weekly telephone phone calls.
**My-GMC** Visser et al. (2018), Netherlands [44]	Video conferencing and tablet-based web-based app (videos, survivorship information) and three web-based support sessions.
**Virtual resiliency program for lymphoma survivors** Perez et al. (2021) [53], USA	Group videoconferencing
**Web based chat group for prostate cancer patients.** Lange et al. (2017) [59], Germany	Online chat program, guided by psychotherapists.
**BounceBack** Nguyen et al. (2022) [57], Canada	Online workbooks and telecoaching
**Online mindfulness based intervention.** Peng et al. (2022), China [45]	Online course delivered in group sessions; assignments for home practice (30 mins per day) accompanied by the recordings of 5P medical app (an app to promote mind and brain health and cultivate happiness)
**ConquerFear‐Group.** Tauber et al. (2023), Denmark [60]	Online delivered group sessions
**“Less fear after cancer”—Guided online primary care intervention** (Luigjes-Huizer etl al 2023), Netherlands [61]	Information videos, exercises, three to five 30-min video calling sessions with a mental health worker

Self-guided interventions generally had lower adherence than guided interventions which highlights the challenges of maintaining engagement in entirely self-guided interventions [62]. Smith et al. [62] noted that some professional facilitation, such as an orientation phone call or follow‐ups to troubleshoot difficulties, might be needed to optimise engagement and benefit, even if it limits scalability somewhat. Some contact with clinicians would also help to spot participants who would benefit from a more intensive FCR intervention. Several reviewed studies sent reminders to participants via email [39, 43, 50, 55] or phone message/call [40, 50,] to provide impetus to interact with the intervention and to provide encouragement about coping with FCR. In CAREST trial, reminders by phone resulted in a higher response than reminders by mail. Notifications of new uploaded content can reach all participants or relevant target groups and prolong the positive effects via remaining interactive [40]. The evidence on effectiveness of this approach is lacking and one review [12] has suggested a future study comparing the effectiveness of intervention with and without such reminders.

Real-world personal preference of participants was important in our reviewed studies, especially when offering and evaluating self-guided interventions. Compen et al. [34] reporting on their BeMind study noted that because one inclusion criterion for their study was the ability and willingness to attend both MBCT and eMBCT, the sampling frame was probably not representative of patients who would prefer eMBCT in clinical practice. Because treatment preference was often positively correlated with treatment outcome, the authors speculated that their RCT underestimated, rather than overestimated, the effects of eMBCT. This is a relevant note for future trial designs, also highlighting that further research is required to understand what drives some participants toward a digital intervention over a face-to-face one. Overall, the results from the BeMind study suggested that although the group-based setting was considered important for mindfulness-based interventions, self-guided eMBCT with limited teacher feedback was also effective because one’s self-efficacy and intrinsic motivation, as well as an attitude of self-discipline, were also important for treatment outcomes.

In the studies that utilised telecoaching, it was reported to increase intervention adherence and engagement, site use and retention [43], to help integrate what the survivors had learned, to provide clarification, and to prepare participants for future scenarios [57]. An initial one to one session with the therapist was employed in the ConquerFear-Group group-based intervention [60] to introduce the FCR model, and discuss possible individual vulnerability factors. The authors did not reflect on the influence of this, but a different study [63] had suggested that alliance building in web-based interventions may enhance engagement to treatment. Martin et al. [52] suggested that integrating a chat function was one way to give survivors the option to reach out to their peer network if desired, but not embedded in the programme as compulsory, which could alienate some potential participants.

Perez et. al. [53] observed a twofold benefit of remote group sessions. First, the changed setting of care by using a videoconference platform for some survivors allowed to avoid immediate negative associations with the site of cancer diagnosis and treatment which would otherwise have deterred them from visiting their cancer centre due to anxiety. Simultaneously, this allowed all survivors to receive support from providers within their own trusted institution, which might provide patients with a sense of care continuity. Thus, it was beneficial to both types of post-treatment patients and is important to consider when preference for a group setting is expressed [49].

Overall, there was a broad consensus amongst the studies that entirely self-managed web-based interventions were not as effective as those supplementing the web-based content with either a one-to-one follow up with nurses, coaches, doctors, access to peer forums, or taking place in a group format [51, 60, 68]. Many recommended adding professional support, like email contact or face‐to‐face assistance to future web-based interventions for FCR.

### Adherence to interventions: rationale and technological capabilities

Higher adherence to web-based interventions had been found to be associated with better outcomes [69], however the evidence in our reviewed studies is mixed. In the FoRtitude study, higher use of the site was associated with greater reduction in FCR, however in the ConquerFear-Group study [60], the number of sessions attended and amount of homework completed were not associated with changes in FCR. hence it is important to ensure that the digitally enabled modality of FCR interventions is not affecting this aspect of the interventions negatively. Matis et al. [12] hypothesised that attrition rate of patients with cancer may be higher in web-based mindfulness-based programs than in face-to-face programs. Where addressed, the adherence and finishing rates in our reviewed studies varied: 44% in BREATH study [33], ‘about half’ in BeMind study [64], 70% in AIM-FBCR [32], 83% in MindOnline [36]. 50% in iHOPE intervention [52]. Peng et al. [45] speculated that the very low dropout rate from their study (three people out of 60) was partially due to a user-friendly app accompanying the web-based group sessions. These overall positive findings contrast with what Lange et al.’s [59] reported about their web-based chat program for prostate cancer patients: only 18 patients (5%) participated in the intervention group till follow-up. The mean age of participants in this study was 60.5 which was the oldest among the reviewed studies, but inferring that these low engagement results were due to lower digital literacy of older participants is not possible. Relevant to this review was Heiniger et al.’s [47] observation that completion rates may not be a good indicator of programme acceptability and feasibility because users of web-based interventions may prioritise accessing only information deemed immediately and personally relevant. Hence, e-TC 2.0, an updated version of e-TC had been made publicly accessible at any time without screening and compulsory modules [48]. This might be a suitable approach to increase engagement in cohorts that are traditionally less likely to take part in various interventions, such as males [47, 59].

One valuable technological capability of web-based interventions is the ability to track website usage [51] and it has been utilised in a small number of our reviewed studies, tracking a number of logins [43], spent using the app [48]. However, some aspects of intervention content were not possible to track, such as meditation practice [39, 64] and were based on self-report, which cannot be held to be objectively accurate. As meditation is an important aspect of any mindfulness intervention, this is an important point for future research on not only mindfulness-based online interventions for FCR, but for the observable and recordable aspects of web-based interventions at large. The ability to personalise some aspects of interface of web-based FCR interventions was touched on in some of our reviewed studies [41, 44] and might provide added benefit, such as increase in exercise completion rates [55]. Also, including personal stories of people with a similar experience can be a way of promoting shared understanding, connectedness and normality [47].

### Longevity of accessibility of resources

Our reviewed studies revealed that the accessibility of intervention materials after the program ends could affect the effectiveness of the intervention. Smith et al. [62] noted that they had originally intended to release iConquerFear website modules every 1 to 2 weeks to encourage skill practice and consolidation, but participants claimed they might disengage if unable to progress at their own pace. While accessing the programs at one’s own pace and convenience was one of the reported attractive features of self-managed web-based interventions there are also several significant limitations to consider. For example, in the BREATH trial [33] attempting to maintain or improve adherence each week, new materials were released periodically, accompanied by standard e-mail reminders. Access to BREATH website was withdrawn after the end of the intervention at 16 weeks which contributed to loss of long-term improvements in psychological distress and FCR. FoRtitude trial [43] also denied access to the FoRtitude website after 4 weeks which, authors speculated, might have been a significant factor for their negative study findings. On the contrary, Fang et al. [41] argued that their positive outcomes, especially the significant decrease in FCR at the 12-month follow-up, might have been substantiated by the fact that their app continued to provide new information during the follow-up period. Overall, the reviewed studies suggested that lasting access to the resources and the ability to actively interact with the content after the intervention concludes was important for web-based interventions that seek to reduce FCR beyond the trial period [51].

### Technical difficulties

Interestingly, only three studies included in our review reported directly on technical difficulties encountered by participants [36, 50, 53], most often access and navigation issues. As many of the studies noted that their sample included populations of relatively high level of education, comments on digital poverty were overall lacking, merely relegated to a limitations section that the sample was not representative of general population. Lack of internet access [40] and access to smartphones [55] were mentioned as problems for rec. Imai et al. [50] drew attention to more rarely addressed aspects of computer, tablet or smartphone-based interventions: increased sleep disturbance, activity reduction, visual loss and bumping into something. Some of these and similar effects are hard to observe and measure. Otherwise, most studies presented recommendations for improvement from participants as they related to the content and presentation of the interventions. These were varied: the need for more resources on lifestyle and family support [51]; more straightforward navigation pathways [51] the need to stay on top of browser upgrades (Heiniger et al. 2017); having a wider representation of cancer survivors in the intervention material to increase relatability [51], creating more mobile friendly websites [51] or mobile apps with a user friendly, visually appealing interface [36].

### Transition to pathways/implementation to care

Only a small number of our reviewed studies had specific comments on this subject. e-TC study [47] included men who had completed treatment for testicular cancer, but the participant feedback was that having access to such an intervention earlier in the illness trajectory would be of greater benefit. iConquerFear [62] participants felt it would be best to offer iConquerFear at completion of active hospital‐based treatment, when decreasing levels of contact with the health system would result in people becoming more fearful. Referral or endorsement from a trusted health professional or organisation might increase uptake and engagement, but the authors noted that more research was needed on how best to disseminate web-based interventions. Indeed, van Helmondt et al. found that recruitment of patients by oncology nurses to the CAREST trial resulted in a higher response than recruitment by mail which indicates the important role usual healthcare practitioner endorsement might possibly play [40]. However, encouraging health care practitioners to refer to programs where they are available is as much of a challenge as making programs available post-evaluation [62]. iCanAdapt-Early [65] programme was placed in primary care pathway across the 12 participating cancer services but the number of referrals made was extremely low and none of the referrals were taken up. Only over a quarter of self-referrals made were within the cut off point for screening for eligibility. In contrast, one study on an intervention in primary care setting [61] used self-referral their recruitment strategy and found that, while the intervention had been designed for patients with moderate FCR, 53% of the group started with high FCR (FCRI-SF ≥22). The same authors have concluded that their FCR intervention in the primary care setting could be sufficient for many patients, especially if there are waiting lists for specialised care. However, for other patients (e.g. those with childhood trauma or other complex needs) it may be better to start with specialised care from the beginning. Thus, the questions about the most suitable strategies of increasing uptake of FCR interventions and during which points in survivorship journeys to introduce them [12] persist.

## Discussion: Knowledge gaps and future directions

Collectively, the reviewed studies, systematic reviews and metareviews on web-based interventions agree that such interventions have potential but require more rigorous, sufficiently sampled trials with larger sample sizes to confirm effectiveness [13, 20]. The reviewed studies also suggested a need for longer term follow-up and outcome measuring period, better defined trial eligibility criteria, more diverse samples regarding sex, education, degrees of digital literacy, income, culture, language and cancer type [51]. There was a concern across the studies that the web-based intervention participants represented a more educated, digitally literate stratum of society. Overall, the reviewed studies suggested that it should be a priority for future interventions for FCR to proactively screen survivors with higher levels of FCR to participate in trials in order to get a convincing account of effectiveness of the intervention in reducing FCR.

This review highlights the complexities regarding the benefits and drawbacks of entirely self-managed interventions. Some features of self-managed interventions, such as the individual nature and the asynchronous interaction were preferred by some patients over a digitally enabled group encounter, which means that the development and proliferation of them would have the potential to engage cancer survivors with lower social skills or anxiety in social settings. The consensus across reviewed studies is that future studies should assess how intervention accessibility, adherence, and effectiveness can be improved in different intervention designs, varying from intensive synchronous individual therapist-assisted web-based programme to blended designs combining the advantages of face-to-face and Internet-based elements, to entirely self-managed programmes. This would help understand the optimal degree of guidance needed and the role of therapeutic alliance [63] in web-based FCR interventions to maximise engagement while minimising cost. There would be value in considering in further research the value of structured psychotherapeutic approaches compared with on-demand accessible support and information tools not only in regard to different patient FCR levels but also in relation to the timepoint in the survivorship journey as the needs of the survivors change with time. Generally, having more robust evidence on which treatment formats provide best outcomes to the reduction of FCR would help influence the design decisions of remotely delivered interventions. While web-based interventions do not involve the costs of transportation, traveling time, space, equipment, cleaning, and other expenses and could be more cost effective, further cost utility analyses should be performed. For example, Lengacher et al. [49] noted that their mMBSR(BC) program had the potential to reach cancer survivors who may be economically disadvantaged and/or underserved to attend a regular MBSR class. However, mMBSR(BC) was delivered through the use of iPad and Lengacher in their paper had not made a note about the provision of these devices to survivors who were not likely to own them if they were economically disadvantaged and the costs involved if providing them. Visser et al. [44] noted that they provided specifically programmed iPads.

Web-based programs aiming to reduce FCR should attempt to make full use of the technological capabilities that allow to create highly usable, flexible, adaptable structures of content and interaction that can be tailored to cater to arising needs during different steps of the cancer journeys. The quality of future feasibility studies of web-based interventions would be improved if they reported in more depth the technological barriers to engagement and the measures taken to overcome them, as well as assessing the interventions through more digitally versatile delivery modes (i.e. application-based, or email/chat communication-aided [39]. Tracking participants’ online behaviour, exercise completion, meditation times, i.e. moving beyond the self-reports of participation, would provide invaluable information on the impact of and preferences for the various aspects for the web-based interventions, which could help to improve engagement, adherence and satisfaction. It would also be worth researching whether intermittent assessment increased adherence. Overall, the reviewed studies revealed that research examining the reasons for dropout would be critical in improving the efficacy of web-based interventions.

Generally, evidence on real world uptake of web-based interventions for FCR is lacking. One way to envisage where web-based interventions for FCR reduction might fit in was in a stepped care model for addressing FCR [54, 60, 62, 70]. Moderators examining the effects of age, education level, and treatment type could also lead to a deeper understanding of which participants would benefit more from such interventions [41]. Further research on how to maximise the uptake and utility of web-based interventions, including determining the optimal points of introducing such interventions in relation to disease trajectory, determining clear referral pathways to the intervention as well as links back to additional services is needed [62], as well as involvement of PPI and underserved populations to better understand the barriers and facilitators to uptake [71]. In addition, further studies could utilize recruitment strategies aimed at widening participation for participants from ethnic minorities and low-income backgrounds.

The decisions on what framework to use for the web-based FCR interventions in our reviewed studies were mainly based on citing that the chosen framework (CBT, ACT, mindfulness, hope theory, etc.) had empirical evidence of effectiveness of decreasing FCR or more generally of improving quality of life and other psychological symptoms in cancer survivors, such as depression or anxiety. One systematic review and meta analysis found the Internet-based mindfulness interventions had no significant effects on fear of cancer recurrence [72]. CBT-based approaches had the largest body of evidence of effectiveness. Generally, the rationale for the included papers was to evaluate whether a web-based intervention based on selected psychological framework was feasible. Hence, from this review it is hard to claim that one psychological framework holds the supremacy over others in tackling FCR. However, what we discovered was that the choice of content of the interventions depended on the intended level of engagement and mode of delivery, e.g. if the intervention was a self-guided app as an addition to standard care, then it appeared that strategies that emphasise individual practice and are based on providing information, such as mindfulness-based or a survivorship plan content would be most suitable; for a clinician-guided course–cognitive and behavioural approaches, such as CBT or ACT might be more suitable as their therapeutic effects are also reliant on group or therapist-patient dynamics. The selection of synchronous vs asynchronous mode of delivery (or a combination of both) would also be influenced by the selected psychological framework.

### Study limitations

This review included only articles written in English and did not consider grey literature, such as doctoral dissertations, thus not all relevant publications might have been included. Another limitation is that the data was searched, extracted and analysed by one reviewer (SZ). However, the search can be reproduced using the search terms, hence is robust enough. The narrative approach to systematising data is reliant on somewhat subjective decisions on what is included in the narrative. However, this fits with the nature of aims of scoping reviews and we do feel that this narrative will be a helpful tool for any readers looking for an easily readable synthesis of existing knowledge about what works and doesn’t work regarding web-based interventions for FCR.

## Conclusion and future directions

Effective FCR treatments need to reflect the multi-dimensional nature of FCR. The studies reviewed here and those being currently trialled [73–78] are based on different psychological frameworks and modes of delivery and provide insight into long lasting, reusable, less labour intensive FCR interventions that can be dotted across various points of survivorship journeys. The exact combinations and ‘doses’ of these different interventions to deliver optimal results are unclear at the moment and will also vary from individual to individual. The continuously growing body of evidence of web-based interventions has the potential to populate the psychosocial cancer care landscape with well-rounded responses to FCR as an unmet cancer survivorship need.

The papers included in this review suggested several key points for future research on web-based interventions for FCR. First, the studies suggested a need for longer term follow-up and post-intervention outcome measuring period and better-defined trial eligibility criteria. They highlighted that it is a priority for interventions for FCR to proactively screen survivors with higher levels of FCR to participate in trials in order to get a convincing composite view of effectiveness of the intervention in reducing FCR. Second, more research examining the reasons for dropout is critical to improve the effectiveness of web-based interventions. Third, while web-based interventions do not involve the costs of transportation, traveling time, space, equipment, cleaning, and other expenses and could be more cost effective, further cost utility analyses should be performed. Fourth, allowing the participants to maintain access to the intervention materials and more so, updating it intermittently after the intervention trial concludes, has a great effect on long-term FCR outcomes. Finally, future studies should assess how intervention accessibility, adherence, and effectiveness can be improved across different intervention designs, varying from intensive synchronous individual therapist-assisted web-based programme to blended designs combining the advantages of face-to-face and Internet-based elements, to entirely self-managed programmes.

Digital interventions have an important and unique capacity to remain accessible, to be constantly updated and evolved without excessive efforts and costs. Overall, there is a much work to be done but also much to be excited about the existing scholarly consensus that web-based interventions have the potential to become a significant tool in onco-psychological care.

## Supporting information

S1 Checklist Prisma checklist. (DOCX)

S1 Appendix (DOCX)
