# Supplementary material for: Web-based interventions for fear of cancer recurrence: A scoping review with a focus on suggestions for the development and evaluation of future interventions
Source: PLoS One. 2024 Nov 8;19(11):e0312769. doi: 10.1371/journal.pone.0312769 (PMC11548736; doi:10.1371/journal.pone.0312769)
Supplement: S1 Appendix — (DOCX) [file pone.0312769.s002.docx]

Appendix 1.

**SEARCH QUERY:**

(fear* OR worr* OR *stress*) AND (recur* OR relaps* OR coming back OR progress* OR *occur* OR spread*) AND (cancer* OR neoplasm* OR tumour* OR tumor*) AND (online OR digital* OR remote* OR virtual* OR cyber* OR computer* OR web OR internet* OR eHealth OR mobile OR tele* OR e-health OR smartphone OR multimodal* OR tablet*) AND (training OR intervention OR help OR therap* OR tool OR strateg* OR manag* OR program* OR platform OR app* OR session* OR support*)
